# Ultra-brief intervention for problem drinkers: three-month follow-up results from a randomized controlled trial

**DOI:** 10.1186/1940-0640-7-S1-A17

**Published:** 2012-10-09

**Authors:** John Cunningham

**Affiliations:** 1 Center for Addiction and Mental Health, Toronto, Ontario, Canada

## 

Helping the large number of problem drinkers who will never seek treatment is a challenging issue. Public health initiatives employing educational materials or mass media campaigns have met with mixed success. However, clinical research has developed effective brief interventions to help problem drinkers. This project employed an intervention that has been validated in clinical settings and then modified into an ultra-brief format suitable for use as a public health intervention. This randomized controlled trial sought to establish the effectiveness of an ultra-brief personalized feedback intervention for problem drinkers. Problem drinkers (N = 1824) recruited via a baseline population telephone survey were randomized to one of three conditions: personalized feedback pamphlet, control pamphlet, or no intervention (control condition). In the week after the baseline survey, households in the two pamphlet conditions were sent their respective pamphlets by mail. Changes in drinking were assessed at three months. The follow-up rate was 83% (1529 participants). Preliminary analysis indicated that there was no significant impact (p > 0.05) of receiving the intervention pamphlet. Although the ultra-brief intervention had demonstrated promising results in two earlier pilot trials, results from the current trial failed to find any impact of this personalized feedback intervention for problem drinkers when delivered to households rather than directly to individuals. Combined with the results from the earlier trials, we surmise that the intervention may have an impact among recipients who voice an interest in receiving such materials but it is ineffective as an unsolicited public health intervention.

